# The Twelve Ds: An Update to Edwards and Benson’s Reasons for Non-Parental Caregiving

**DOI:** 10.3390/ijerph20095618

**Published:** 2023-04-24

**Authors:** Acacia R. Lopez, Danielle K. Nadorff, Delaney Peters

**Affiliations:** Department of Psychology, Mississippi State University, Starkville, MS 39762, USA

**Keywords:** grandparents raising grandchildren, foster parents, reasons, caregiving

## Abstract

This qualitative study examined the prevalence of the “Nine Ds,” a framework developed by Edwards and Benson for understanding the heterogeneity of reasons for which grandparents assume care of grandchildren (i.e., death, disease, detention, divorce, departure, drugs, desertion, delivery, deployment) in a contemporary sample. Using a nationwide sample of custodial grandparents (*N* = 322) and foster parents *(N* = 105)*,* caregivers were asked their reason for assuming care of the grandchild or foster child within their care. The results of the study suggest that the Nine Ds are a useful framework, but accounted for only 21.74% of responses, indicating the Nine Ds fail to capture many of the reasons for assuming care. Three new themes—dollars, duty, and daily grind—were identified using semantic thematic analysis and are applicable to both grandfamilies and foster families. These themes represent different motivations for assuming care and provide insight into the social structures that may act as barriers to family formation. This study provides a foundation for future research examining the impact of assumed care by non-parental attachment figures on the health and well-being of both grandchildren and foster children.

## 1. Introduction

In 2021, there were approximately 7,094,232 grandparents living with their grandchildren under the age of 18 in the United States. Of these grandparents, approximately 33% were directly responsible for their grandchildren [1]. “Grandfamilies,” in which grandchildren reside with and receive care from their grandparents, are typically formed as an upshot of unfavorable circumstances in which the child’s parents are unwilling or unable to provide care [2]. The reasons for which a grandparent assumes care of a grandchild are largely heterogeneous, with both voluntary and involuntary decisions by a parent to relinquish caregiver responsibilities [3], and this caregiving relationship may have a significant impact on both the grandparents’ and grandchildren’s health [4]. 

### 1.1. Nine Ds

A survey of the extant literature on the history of grandfamilies’ formation better describes the heterogeneity of reasons to assume care in this population. Four preliminary reasons for which a parent would voluntarily surrender a child to the care of a grandparent were hypothesized by DeToledo and Brown: divorce, desertion, drugs, and death [5]. Further reasons were later identified by Glass and Huneycutt [6], which Edwards and Benson succinctly coined as the “Nine Ds,” [3]: divorce, drugs, desertion, disease, departure, death, delivery, detention, and deployment. The distinction of these additional categories by Edwards and Benson was intended to provide clarification on grandfamilies and their needs as they may relate to prevention and intervention services. The Nine Ds aptly described many of the reasons cited by grandparents for assuming care of their grandchildren and are further reviewed:

- *Divorce.* According to the Centers for Disease Control and Prevention, the most recent divorce rate was 6 per 1000 of the total population [7]. Due to the negative factors such as marital stress, financial instability, and aggressive disputes often present during a divorce, parents may ask their children’s grandparents to care for the children. While the literature supports divorce as one of the nine reasons for grandparents assuming care, it is worth noting that the divorce rate is lower as of 2016 compared to a decade prior, which may affect the overall prevalence of current and future grandfamilies forming as a result of divorce. A possible explanation of the lower divorce rates are lower annual marriage rates, which are projected to continue declining [8]. Original research by Shin and colleagues evaluated adolescent adjustment after parental divorce, specifically evaluating grandparent caregivers who raised their grandchildren. Their study found that grandparents were more likely to seek and accept help from outside the family to aid in burdens associated with caregiving [9].
- *Drugs*. Parental drug use can lead to a child being voluntarily or involuntarily placed in the grandparent’s home. The connection between drug addiction and grandfamilies has received particular attention in the literature as a result of the opioid epidemic, which was declared a national public health emergency in 2017 by the U.S. Department of Health and Human Services. There has been an increase in the number of children living with grandparents, and furthermore, it was found that the states with the most grandfamilies also had the highest opioid prescription numbers [10,11]. A qualitative study of 15 grandparents who were raising their grandchildren as a result of the opioid epidemic showed that existing prevention and intervention services were not adequate in meeting grandparent caregiver needs as they related to legal, financial, emotional, and family needs [12].
- *Desertion.* When the child is abandoned by their parents, often due to abuse or neglect, and the grandparent takes responsibility for raising them. This may take the form of children being sent to live with their grandparents after an abuse report is filed with a state welfare agency, such as Child Protective Services [2].
- *Disease.* Debilitating diseases can cause parents to be unable to care for their child. Historically, the literature has highlighted the impacts of a parent having significant health crises, such as cancer and AIDS, as main reasons for grandparents to assume care of their grandchildren [3]. Other examples of diseases may include physical illnesses such as multiple sclerosis or severe mental illnesses [13]. Another significant health crisis that may impact the future prevalence of disease as a differential reason for care is COVID-19, which has affected millions of individuals around the globe [14]. Original qualitative research on grandparents who raised grandchildren as a result of the COVID-19 pandemic found that both grandparents and grandchildren had increased feelings of isolation. Zakari and colleagues called for telehealth support services to minimize feelings of self-isolation within this population [15].
- *Departure.* Most common in impoverished countries or regions where resources and opportunities are scarce, parents may choose to migrate to a developed country to find better access to employment. Children are left in the care of grandparents during the parent’s departure [3]. This may also include families who have been impacted by immigration issues, such as deportation. A study by Beltran and Cooper found that these grandfamilies face barriers related to the immigration status of the child, which may impact the ability to apply for guardianship, enroll the child in school, and consent to health care choices for the child [16].
- *Death.* Grandparents may be inclined to assume the care of a grandchild when a child’s parent dies. Specifically, studies cite that grandparents choose to assume care in order to avoid foster care for the grandchild [17]. Research on children who have experienced the loss of a parent show that they are in significant need of support. Grandfamilies are affected by the death of a parent in unique ways, as both the grandparent and the grandchild are mourning the loss the of the child’s parent. These grandfamilies may have an increased need for grief counseling and support services compared to other grandfamilies [18].
- *Delivery.* When adolescents give birth, many are unprepared to enter into the responsibilities of parenthood, causing grandparents to assume care for the infant. In 2017, the CDC estimated a birth rate of 18.8 per 1000 women aged 15–19 in the United States, totaling 194,377 infants born to adolescents. This statistic, while dropping, may have a salient impact on differential reasons for assuming care. Bailey and colleagues analyzed the lived experiences of 26 grandparent caregivers and the burdens they faced. For those who were raising grandchildren as a result of a teenage pregnancy, they reported increased financial strain due to a number of unexpected expenses. Many caregivers stated they had to delay their retirement as a result [19].
- *Detention.* Parents who are in jail or prison are unable to care for their child, and a new guardian is needed while they are incarcerated. Based on a 2016 survey of prison inmates, an estimated 1.5 million children (under the age of 17) had a parent who was in either a state or federal prison, potentially causing grandparents to assume care of the grandchild [20]. A recent study found that over 1000 people give birth in prison each year, and identified grandparents as the most common caregiver for these children [21].
- *Deployment.* Children of military members may be cared for by a grandparent if the parent is deployed for war. Historically, large wars have been emphasized as a reason for grandparents to assume care of their grandchildren, such as Operation Iraqi Freedom [22]. The results from a qualitative study on the lived experiences of 23 grandparent caregivers due to deployment found that these grandparents face significant challenges in their intimate and social relationships and have high levels of stress, which may impact the grandchildren for which they care [22].

The Nine Ds cited by Edwards and Benson are applicable to various types of grandparenting households, including those in which a parent lives in the same household (“multi-generational households”) and those in which the parent does not (“skipped-generation households”). Reflective of current census data, parents may live in the same household as the grandparents and children, but many caregiving responsibilities are surrendered to the grandparent. Furthermore, Edwards and Benson argue that the phenomenon of grandparents raising grandchildren is pervasive across many different strata, including SES, race, and geographic location, suggesting that reasons for assuming care should be robust amongst these demographic variables [3].

While grandchildren raised by their grandparents represent a large number of children whose parents were unwilling or unable to care for them, they are not the only group with this background. Scenarios in which children who are removed from an unfit home by a child welfare agency (i.e., a department of Child Protective Services) often result in these children entering into the foster care system. In 2021, there were 391,098 children reported to be in foster care [23] (44% in non-kin home care, 35% in kin care).

Although there are far fewer children in the US being raised in foster care than those being raised by their grandparents, the two groups are similar in many ways. For example, they share many of the same reasons for leaving their parents’ care, as well as similar outcomes. Children are raised by their grandparents or within the foster system as a result of their parent being unwilling or unable to care for them. The most common reasons for children to be placed in foster care were parental neglect (63%) and parental drug abuse (34%), which are also identified in the Nine Ds (desertion and drugs). Children in both groups also share increased vulnerabilities for psychopathology, including both internalizing and externalizing behaviors [24]. For these reasons, children raised in foster care provide an excellent comparison group for custodial grandchildren, rather than children raised in a two-parent household, who have historically served as the usual comparison group in the extant literature.

The previous literature has called for understanding the heterogeneity of families in which grandparents care for the grandchild, including the contexts of and reasons for assumptions of care. Despite being a large population, research on the reasons for which grandparents choose to care for their grandchildren is based on original research on specific contexts, such as incarceration or substance use [10]. Furthermore, there are no studies that investigate the prevalence of all nine reasons described by Edwards and Benson across a nationwide sample [3]. Thus, there is limited understanding of the most common reasons for which grandparents assume care of their grandchildren.

Clarity on reasons for assumptions of care may provide insight into specific areas of concern for grandchildren raised by grandparents, including increased risk for psychopathologies, such as depression and anxiety, and potential impacts on the parenting abilities of the grandparent related to the reason for assumption of care [3,25,26]. Specific types of separation from a parent may also result in different types of disruptions in attachment. For example, parental incarceration and death would cause temporary and permanent disruptions in the parent–child dyad, respectively [27]. Additionally, a better understanding of the background and history of families would help to provide a framework that could be utilized to help children, parents, foster parents, or grandparents prevent and cope with associated difficulties. A comparison between reasons for assumption of care by grandparents and foster parents would also allow for identification of both strengths and areas of improvements in kin care, including needs relating to prevention and intervention services.

### 1.2. Current Study

A review of the grandparenting literature by Edwards and Benson synthesized original research to identify nine main reasons (“Nine Ds”) for which grandparents assume care of their grandchildren. However, to our knowledge, there are no studies that evaluate all nine reasons in one study to assess prevalence [3]. Therefore, it is unclear which reasons are most common for the formation of this non-traditional family type. Additionally, while the foster care literature is replete with studies documenting the circumstances and prevalence for which a child enters foster care, there is little research documenting differences between custodial grandparents and foster parents who choose to care for children. Thus, this study aims to assess the prevalence of reasons for care in a national sample. Understanding of the prevalence in a national sample may contribute to more tailored prevention and intervention services for this population of caregivers and children. Accordingly, the goals for the present study were two-fold:

1. Determine the prevalence of reasons for assumed care by grandparents in a nationwide sample based on the Nine Ds framework outlined by Edwards and Benson, and assess if the nine reasons posited by Edwards and Benson capture the majority of responses in a population of grandparent caregivers.

2. Compare the reasons for which individuals assume care for children between grandparents and foster parents.

## 2. Materials and Methods

### 2.1. Participants

A nationwide sample of custodial grandparents and foster parents (*N* = 427) in the United States was recruited online using Qualtrics Panel Service (QPS). QPS maintains a pool of people who participate in research opportunities for payment on an ongoing basis and are then recruited for eligible studies based on inclusion criteria set forth by each study. Compensation for participation in research is provided directly by QPS and exact amounts are not disclosed to researchers. Qualtrics utilizes a double opt-in and identity verification process to ensure the validity of demographic information from participants [28]. This type of sampling has been found to be demographically representative and reliable for survey-based research [29]. To assess eligibility for this study, participants were screened using a double opt-in and identification process and were required to endorse caring for either a grandchild or foster child by answering “Yes” to one of the following statements: “My grandchild lives with me” or “At least one foster child lives with me.” Participants who did not report a grandchild or foster child currently residing with them were ineligible for the study. Further eligibility criteria included: 1) must be able to read and write in English and 2) must be 18 years or older. Eligible participants were provided with informed consent forms and were able to withdraw from the study at any time. Participants were treated in compliance with ethical procedures as outlined by the American Psychological Association and the Declaration of Helsinki.

The recruited custodial grandparents (*N* = 322) were United States residents and ranged in age from 27 to 84 years (*M* age = 55.66 years, *SD* = 9.52). Custodial grandparents self-identified as white (85.1%), African American or Black (10.2%), biracial or multiracial (1.9%) and other (2.8%). A large majority of custodial grandparents in this study were female (86.1%), and about half reported being married (52.3%).

The recruited foster parents (*N* = 105) were United States residents and ranged in age from 18 to 61 years (*M* age = 34.45 years, *SD* = 7.95). Foster parents self-identified as white (77.1%), African American or Black (8.6%), biracial or multiracial (9.5%) and other (4.8%). Foster parents were predominantly female (77.1%), and 78.1% reported being married. More specific demographic details for both foster parents and custodial grandparents are presented in Table 1.

### 2.2. Measures

Participants completed a demographic questionnaire, and reasons for care were assessed using the open-ended questions “Why did you choose to raise your grandchild(ren)?” and “Why did you choose to become a foster parent?” for grandparents and foster parents, respectively. Participants answered by typing their answers into the online survey platform. Responses had no word or character limit.

### 2.3. Procedures

Participants were screened online to determine their caregiving status. Eligible participants were given informed consent forms and subsequently completed an online survey. Participants’ responses were then analyzed and coded by two independent coders according to the *Nine Ds* framework. Responses that were not able to be coded according to the *Nine Ds* framework were subsequently analyzed using semantic thematic analysis, a method outlined by Braun and Clark as appropriate for describing themes in qualitative data [30]. Interrater reliability was established for the raters based on recommendations from Zegers et al. [31]. Comparison between caregiver groups was achieved by examining the distribution of codes in each group, a technique that is consistent in the qualitative comparison literature [32].

### 2.4. Raters

Two independent raters were used for this study (*M* age = 23.5). Both raters had educational backgrounds in psychology; one rater was a senior psychology undergraduate student and the other was a first-year clinical psychology doctoral student. Raters had differing ethnic backgrounds. Both raters were familiarized with the *Nine Ds* framework before coding responses.

## 3. Results

SPSS 28.0 (IBM Corp., Armonk, NY, USA) was used to determine the interrater reliability of agreement between the two raters. The overall interrater agreement was high, κ = 0.94, 95% CI [0.91, 0.97]. Zegers and colleagues state that values of interrater reliability ranging from 0.81 to 1.00 suggest “almost perfect,” or high agreement between raters [31]. Interrater reliability is provided for each reason for assumption of care in Table 2.

The Nine Ds accounted for approximately 21.74% of reasons for which grandparents assumed care of their grandchildren and 3.81% of reasons for which foster parents assumed a caregiver role. Of the Nine Ds, the most frequently cited reasons for grandparents to assume care included desertion, drugs, and divorce. Departure was the only reason of the Nine Ds not reported in this sample. Foster parents reported only three of the Nine Ds: death, disease, and detention.

The remaining responses that were not classified by one of the Nine Ds were analyzed for identifiable patterns using a semantic thematic analysis approach, as outlined by Braun and Clarke [30]. Semantic thematic analysis allows for a theoretical identification of patterns from the lived experiences of participants, which are inferred by raters from the written response of participants, and results in themes. This level of identification was appropriate as researchers were interested in the explicit reasons caregivers articulated for choosing to care for children. Three new identified themes emerged from the semantic thematic analysis: dollars, daily grind, and duty. These new themes accounted for approximately 63.89% of total grandparent reasons and 76.24% of total foster parent reasons. See Table 3 for a summary of reasons for assumption of care reported by both grandparents and foster parents. Separate consideration of each of the new themes is presented.

Separate consideration of each of the new themes is presented. For illustrative purposes, quotes from caregivers are provided to highlight the associated meanings of the themes. Each participant quote is followed by demographic details: FP = foster parent or CGP = custodial grandparent; F = female or M = male; age.

### 3.1. Dollars (2.56%)

Dollars was an identified theme for custodial grandparents whose own children faced financial strain or burden. Financial burden prevented access to daycare in many cases, resulting in grandparents providing the majority of care for the grandchildren. Additionally, this theme represented those who stated that their children were not financially independent or stable and, therefore, did not have the means to support the basic needs of a child. Most, but not all, cases explicitly stated that both the grandchild and the child (middle generation) lived within the same household.

Example quotes:

Because [my grandchild’s] parent cannot afford to raise them by themselves. [CGP, F, 56]
My daughter needed our help, financially. [CGP, F, 49]
To help offset childcare for my son (my grandson’s dad). [CGP, F, 57]
To help their parents, who are both unemployed. [FP, F, 57]

### 3.2. Daily Grind (4.81%)

Daily grind described custodial grandparents who cared for their grandchildren in order to allow the parent of the child (middle generation) to work or attend school. Some of the grandparents indicated that they cared for the grandchild only during work or school hours, and that the grandchild did not reside with the grandparent full-time.

Example quotes:

I am not raising my grandchild all by myself. My daughter also lives with us. I only watch [my grandchild] during the day so she can work. [CGP, F, 52]
I’m helping to raise [my granddaughter] while her mother goes to school and her father drives a truck. [CGP, F, 71]
I’m helping her mom! She is working full time and I help her with raising. The dad isn’t involved. [CGP, F, 44]
My children work. [CGP, M, 71]

### 3.3. Duty (68.91%)

Duty involved a range of associated meanings for both grandparents and foster parents. Many grandparents reported feeling an inherent sense of responsibility to care for their grandchildren so their grandchildren would not become wards of the state. Others reported feeling a strong need to help their own children by caring for their grandchildren.

Foster parents reported an onus to care as well. Many responses reflected a desire to “give back,” as the foster parent was once a child within the welfare system themselves. In sum, this underlying theme was best represented by emotional and inherent obligations to care for children in both foster parents and grandparents.

Example quotes:

I didn’t want them to be put in the system. They are the apples of my eyes and I don’t want them to be cared for by anyone but family! [CGP, F, 50]
Because I love my son and my grandchildren. I don’t want [my son] to lose his children. [CGP, F, 48]
It was the right thing to do. [CGP, F, 64]
Family is very important. Blood takes care of blood. You do what you must. [CGP, F, 61]
I love them. It’s my responsibility to do the best I can for my children and grandchildren. [CGP, F, 65]
I was a child in [foster]care and I wanted to give back and adopt. [FP, F, 29]
I was a foster kid and I wanted to give back. [FP, F, 35]
To make a difference. [FP, M, 45]

### 3.4. Note on Foster Parents

While the three new themes accounted for a large majority of the remaining responses, there were some responses that were not adequately captured by these themes. Many such responses were specific to foster parents, who indicated choosing to assume a caregiver role for reasons related to the inability to conceive naturally or due to fertility complications, or were members of the LGBTQ community who wished to raise a family.

Example quotes:

Because I’ve always been interested in having kids but cannot have any of my own. [FP, F, 20]
Because I couldn’t get pregnant. [FP, F, 38]
My husband and I wanted a child and because we are both males, we wanted to adopt a child. [FP, M, 34]

## 4. Discussion

The Nine Ds were originally identified by Edwards and Benson and developed from a review of the grandfamily formation literature. A review of the literature revealed that the prevalence of these reasons had not been assessed in a nationwide sample of grandparent caregivers. Thus, the first goal of the present study was to identify the prevalence of the Nine Ds in a national sample of grandparent caregivers. In this study, grandparent caregivers reported eight of the nine reasons cited by Edwards and Benson, with the most frequent reasons being desertion (6.70%) and drugs (5.36%). In contrast to the study by Edwards and Benson, departure was not endorsed as a reason for care in this study. A possible explanation for why this reason was not identified is due to the geographic sampling of the current study. Departure is defined as leaving unpropitious working conditions to seek opportunities in developed countries, such as New Zealand and the United States. Given that the sample is from the United States, this category may not be as prominent as regions in which departure is frequently seen, such as South Asia.

While the present study was able to successfully identify reasons for care consistent with the Nine Ds in a nationwide sample, the Nine Ds did not account for a large majority of the responses from grandparents. Approximately 83.43% of the reasons provided by grandparents were not able to be categorized by one of the Nine Ds, suggesting that new themes may be necessary to explain the remaining reasons for which a grandparent assumes care. Using semantic thematic analysis techniques, three new themes emerged in the current study to explain reasons for care: dollars (3.46%), daily grind (6.49%), and duty (59.74%).

The results of the three new themes from this study provide both useful insight into motivations for forming non-traditional families and foundational understanding of the types of barriers that non-traditional families may face. The theme of duty provides evidence for motivation behind why grandparents choose to become caregivers to children, as they report feeling an inherent sense of responsibility to care for their grandchildren rooted in family unit preservation. This theme is defined by an innate drive to form and maintain families.

The theme of daily grind reflects caregiving by grandparents as a result of changing societal norms where an increased prevalence of parents, especially women, work a full-time career while raising children. Dual-income households, in which both parents work full-time, are more common now than in previous years [33]. This increase may predict an increased need for grandparents to care for their grandchildren in the future.

The final new theme, dollars, points to social and economical factors that drive the formation of grandfamilies. Parents who are not able to afford basic necessities, such as rent, groceries, or medical care for their child, may be more likely to have the child’s grandparent assist or assume care. This theme represents an important portion of a population who may need increased services, such as education about community programs and services that may assist them in accessing free health care, food stamps, or even job fairs.

Although the new themes are derived from a national sample, grandparent caregivers are a largely heterogeneous population due to the many dimensions of diversity of caregivers, such as socioeconomic status, education level, age, gender, and culture [3]. This characteristic of the grandparent caregiving population has been noted throughout the literature as a significant challenge in studying various outcomes. African American and Hispanic grandparents are disproportionately represented compared to their white counterparts [34]. Socioeconomic status is often correlated with the overrepresentation of minority populations as caregivers, with grandparent caregivers having a lower SES than their same-age peers [35]. As Hayslip and colleagues point out in their comprehensive review of the grandparenting literature, African American grandparents are more likely to take in their grandchildren for longer periods of time, which poses a significant financial strain and may lead to more chronic poverty rates [34]. A majority of the participants in this study self-identified as white, suggesting that grandparents who were of racial and ethnic minorities may not be well represented in this study. This may, in part, be due to the fact that the present study was conducted using an online survey platform. Grandparents with a lower socio-economic status may not have reliable internet or may be required to work to supplement their finances and, thus, may not have time to complete online surveys. While it is a limitation, it also provides a guide point for future research to evaluate how mental health systems and professionals can better assist this group. Community prevention and intervention efforts may need to be integrated with existing systems that grandparent caregivers are already utilizing.

The results also suggest beliefs and values that may be shared across many levels of diversity and that represent cultural norms of the grandparent caregiving population. More than half of the grandparent caregivers in this sample reported “duty” as their primary motivation to care for their grandchildren. This represents a significant shared value of family importance and the belief that the larger family unit should be maintained and protected at any cost. This shared belief transcends many of the diversity factors previously mentioned, suggesting that individuals who work with this population in a prevention or intervention setting as a clinician should develop competencies in the grandparenting culture, in addition to the competencies necessary for other multicultural factors that may be relevant to grandparents, such as race or ethnicity. Sue and Sue’s multidimensional model for developing cultural competencies may provide a useful framework to explore grandparent caregiving competencies. To effectively work with this population, mental health professionals are encouraged to develop a knowledge of grandparent caregiving in general, including the reasons for care and beliefs and attitudes of grandparent caregivers, and skills to work with this unique population [36]. To fully consider Sue and Sue’s model, the competent mental health professional should consider how knowledge of the grandparent caregiving population intersects with individual, professional, organizational, and societal factors that grandparent caregivers may face [36]. Mental health workers who work with this population would need to have knowledge of the various reasons for which grandparents may care for their grandchildren and how this may intersect with other multicultural factors. For example, a grandparent who is caring for their grandchild as a result of the opioid epidemic may face significantly different barriers than a grandparent who cares for their grandchild while the biological parent gains more advanced education. In the former scenario, grandparent caregivers may face barriers at a societal level if the biological parent is either in a substance abuse rehabilitation program or has passed away as a result of substance use, thus being unable to engage in the child’s life. The grandparents may not be able to access health care services for their grandchild if guardianship has not been legally granted [37]. In the latter scenario, the child’s biological parents may still be involved in the child’s life and able to participate more fully in providing for the child, including assisting in health care decisions.

Finally, grandparent caregivers themselves are diverse individuals who will likely have multiple points of intersectionality with their identity as a grandparent caregiver. The competent mental health professional will exhibit knowledge of identity intersectionality within this population. This includes an understanding that the majority of grandparent caregivers are African American or Hispanic women with a low socioeconomic status [35].

This expansion of reasons for assuming care, from Nine Ds to twelve, allows for a better understanding of the reasons for which grandparents care for their grandchildren. Specifically, the identification of the “duty” theme, which accounted for more than half of all grandparents’ reasons for assuming care, provides a better insight into the motivations for caring for grandchildren. Relative to other themes, grandparents were much more likely to identify duty as their primary reason for assuming care of their grandchildren, as evidenced by responses that emphasize an inherent responsibility to keep family together. This is further supported by theory, suggesting that grandparents assume care of their grandchildren in an effort to maintain family identity by not disrupting the family unit, which would be a possibility if the grandchild were to enter into non-kin foster care [38]. By maintaining the family unit and, thus, familial bonds, grandchildren in these families likely have a greater sense of belonging, which has been connected to positive mental health outcomes [39]. This theme informs prevention and intervention efforts by highlighting the need for grandparent caregivers to access more resources that would prevent their grandchildren from entering the foster care system. The previous literature states that grandchildren raised by their grandparent have better outcomes than their same-age peers in the foster care system, and results from this study add to the literature by demonstrating the strong motivation of grandparents to provide a stable family unit for their grandchildren at any cost necessary [40]. This suggests that efforts to seek improved resources for grandparents to aid in the financial and emotional burdens associated with caregiving should continue.

The second goal of this study was to include a similar comparison group, foster families, to determine the Nine Ds’ applicability to and prevalence in multiple types of non-traditional families. Comparative analysis methods across groups in qualitative research, as outlined by Lindsay, were utilized. As such, reasons for care across all caregivers were assessed first, before separating by caregiver type to be compared across groups [32]. The most common reasons within the original Nine Ds endorsed by foster parents were death, disease, and detention. These categories were saturated with responses from foster parents who were also kin (e.g., “My niece’s mother is incarcerated”, “I’m providing kinship guardianship of my niece and nephew who were placed in state care”). These responses suggest evidence of relatives who opted to be caregivers through a formal arrangement with a state welfare agency, rather than many custodial grandparents who informally care for their grandchildren. Similar to grandparent caregivers, the inclusion of the theme “duty” accounted for a large majority of the reasons that foster parents chose to assume care of a child in foster care. Unlike grandparents, however, the sense of duty reported by foster parents was not in an effort to maintain a family identity. Rather, the sense of duty appeared to be driven by efforts of altruism, and for the “greater good”. For example, many foster parents reported that they wanted to “give back” or “just wanted to help.” The nuances in types of duty within both family types suggest that grandparent caregivers and foster parents may have different characteristics. That is, foster parents may be more eager to engage in the caregiver process to achieve a sense of contribution to the greater good, whereas grandparents may face a more urgent sense of duty tied to stabilizing their family unit. This may point to differences in the types of stress faced by each family unit. However, it is important to note that grandparents, although perhaps motivated by a sense of urgency, also hope to contribute positively to their grandchildren and their family unit.

## 5. Limitations and Future Directions

The primary limitation of this study was the self-reported nature of the questionnaires. While the self-report measure did provide an option to write in a response, it did not allow for an interviewer or researcher to follow-up for clarifications. For example, an underlying theme of wanting to “help” emerged for many caregivers. While this does give an insight into the motivations behind a grandparent’s or foster parent’s choice to care for a child, it does not provide clarification on the reasons for which help was needed—perhaps help was needed due to financial strain. That is, it is possible that the new category “duty” is a present and underlying constant in each of other reasons to assume care. Duty perhaps better reflects the motivation of the caregivers, rather than the specific reason a parent was unable or unwilling to care for their child. Future research to clarify the reasons for care should seek to have semi-structured interviews in which a researcher is able to inquire more about particular responses. Additionally, the study had only two independent raters. Future studies would benefit from multiple coders to ensure accurate agreements and reliabilities.

While the three newly added themes dollars, duty, and daily grind were able to identify previously uncategorized responses, future studies may seek to explore other reasons for care that may be more specific. Historical cohort effects may also have an impact on the results. Although these data were collected before its emergence in 2019, the recent public health emergency of COVID-19 has had devastating death tolls, with potential implications for non-traditional family formation such as foster families and grandfamilies. For example, individuals who were furloughed or unemployed during the pandemic may now be members of a multigenerational home.

Future studies should also seek to understand if and how the heterogeneity of reasons for family formation impact the psychological and physical well-being of children, parents, or grandparents. For example, do children raised by grandparents as a result of their parent’s death differ emotionally from children whose parents are incarcerated? As previously discussed, research on this topic would greatly benefit from larger sample sizes, which would allow comparisons across different multicultural factors, including race, ethnicity, gender, and SES. This would allow for a clearer understanding of how resources may be allocated for grandparent caregivers. Additionally, this population may be especially well served by initiatives that provide access to basic necessities, including food, water, housing, and medical care. Future studies that explore the implementation of public programming, including mental health care for grandfamilies, would be especially informative.

## 6. Conclusions

In summary, non-parental caregivers represent a large proportion of those caring for children within the United States, and these caregivers, especially custodial grandparents, assume care of these children for both voluntary and involuntary reasons. The heterogeneity of reasons for which custodial grandparents assume care of their grandchildren was highlighted by Edwards and Benson (2010), who identified nine primary reasons for which grandparents care for grandchildren: death, disease, detention, divorce, departure, drugs, desertion, delivery, and deployment. In the current study, we used a sample of 427 custodial grandparents and foster parents to evaluate the applicability of Edwards and Benson’s Nine Ds theory in a contemporary sample of not only grandparents raising their grandchildren, but also foster parents (two types of non-parental caregivers with many shared antecedents and outcomes). Our study provides a comprehensive overview of reasons for which custodial grandparents and foster parents assume care of a child.

The nine Ds provided by Edwards and Benson were found to be applicable to both custodial grandparents and foster parents within the contemporary sample. However, many additional reasons for care remained after classifying into the nine categories used in Edwards and Benson’s model. Trained researchers used semantic thematic analysis [30] to identify three new themes, which provided new insight into the motivations and social barriers for forming non-traditional families: “duty”, “daily grind”, and “dollars.” Grandfamilies and foster families have many similarities, yet their reasons for care differ in terms of motivation and social barriers. The identification of the new themes reveals the complexity of caregiving families, providing an opportunity for improved services and support for these at-risk families.

## Figures and Tables

**Table 1:** Sample demographic characteristics.

	Foster Parents			Custodial Grandparents		
	*M*	*SD*	*%*	*M*	*SD*	*%*
Difficulty paying bills (1 = Not much difficulty, 4 = A lot of difficulty)	3.10	0.91		2.63	1.04	
Highest level of education						
*High School Diploma or GED*			17.1			41.9
*Associate Degree or Vocational License*			28.6			29.8
*Bachelor’s Degree*			25.7			17.7
*Master’s Degree*			20.0			5.0
*Doctorate or Professional Degree*			6.7			0.3
*None*			1.9			5.3
Marital status						
*Married*			78.1			52.3
*Widowed*			1.0			10.5
*Divorced*			1.9			25.1
*Single, never married*			12.4			6.2
Number of children under care	1.34	0.65	-	0.43	0.74	-
Child age	7.86	4.14	-	8.25	5.04	-
Child gender						
*Male*	-	-	55.2	-	-	51.4
*Female*	-	-	44.8	-	-	47.1

**Table 2:** Interrater reliability by reason for care.

Reason for Care	*κ*	95% Confidence Interval	Agreement
Death	0.86	0.70–0.96	High
Disease	1.00	0.90–1.10	High
Detention	0.89	0.78–0.99	High
Divorce	0.95	085–1.06	High
Drugs	0.91	0.80–1.01	High
Desertion	1.00	0.90–1.10	High
Delivery	1.00	0.90–1.10	High
Deployment	0.86	0.75–0.96	High
Dollars	1.00	0.90–1.10	High
Daily Grind	1.00	0.90–1.10	High
Duty	0.99	0.88-1.09	High

*Note:* Agreement descriptors based on recommendations from Zegers et al. [31].

**Table 3:** Grandparents’ and foster parents’ reported reasons for assuming care.

Reason for Care	Grandparents		Foster Parents	
	Frequency	%	Frequency	%
Death	6	2.60	1	1.24
Disease	7	3.03	1	0.62
Detention	8	3.46	2	1.25
Divorce	11	4.77	0	0
Departure	0	0	0	0
Drugs	11	4.76	0	0
Desertion	15	6.49	0	0
Delivery	8	3.46	0	0
Deployment	4	1.73	0	0
Duty	138	59.74	77	48.73
Dollars	8	3.46	0	0
Daily Grind	15	6.49	0	0
Total	231	71.74	81	77.14

*Note:* The above totals are based on the average between the two coders, with decimal values rounded.

## Data Availability

Data used in this publication are available upon request.
